# A comparison of different established and novel methods to determine horses' laterality and their relation to rein tension

**DOI:** 10.3389/fvets.2022.789260

**Published:** 2022-09-15

**Authors:** Sandra Kuhnke, Uta König von Borstel

**Affiliations:** ^1^Department of Animal Breeding, Kassel University, Kassel, Germany; ^2^Department of Animal Breeding and Genetics, Group Animal Husbandry, Behaviour and Welfare, Justus-Liebig-University Giessen, Giessen, Germany; ^3^Department of Animal Breeding and Genetics, University of Gottingen, Gottingen, Germany

**Keywords:** laterality, horse, rider, rein tension, symmetry, lateral displacement of hindquarters

## Abstract

The present study aimed to assess an agreement between established and novel methods to determine laterality and to identify the distribution of laterality in warmbloods and Thoroughbreds. Nine different methods to investigate a horses' laterality outside a riding context and during riding were compared across two groups of horses (sample A: 67 warmblood- type horses, sample B: 61 Thoroughbreds). Agreement between any two methods was assessed by calculating Cohen's kappa with McNemar's test or Bowker's Test of Symmetry, and the deviation from equal distributions was assessed with chi^2^-tests. Continuous variables such as rein tension parameters were analyzed using ANOVA or linear mixed models. Generally, laterality test results obtained outside a riding context did not agree with laterality during riding or among each other (Bonferroni corrected *p* > 0.0018). However, the rider's assessment of her/his horse's laterality allowed conclusions on rein tension symmetry (*p* = 0.003), and it also agreed substantially with the lateral displacement of the hindquarters (*p* = 0.0003), a method that was newly developed in the present study. The majority of warmbloods had their hindquarters displaced to the right (73.1%, X^2^ = 14.3; *p* < 0.0001). The pattern of lateral displacement of the hindquarters was similar in the Thoroughbred sample (right: 60.7%, left: 39.3%), but did not deviate significantly from an equal distribution (X^2^ = 2.8; *p* > 0.05). Laterality seems to be manifested in different ways, which generally are not related to each other. Attention should be paid to the desired information when selecting methods for the assessment of laterality. Horses' laterality has an impact on the magnitude and symmetry of rein tension. Matching horses and riders according to their laterality might be beneficial for the stability of rein tension and thus improve training.

## Introduction

In several highly evolved mammals, one side of the bilaterally largely symmetric body is stronger developed than the other, be it with regard to bone measurements, muscle development, vascularisation, or—most important—the neural system including the brain (1–5). All these asymmetries of the principally bilaterally symmetric locomotor apparatus and nervous system are usually subsumed under the term “laterality” [see (6) for a detailed review of this topic]. The existence of laterality has been observed in many species including humans and horses for a longtime (2, 7–10). The majority of humans are proven to be right-handed, that is, they are lateralized to prefer their right hand for reaching tasks or using tools (11). Different regions of the brain are responsible for different tasks (3). Dominant limbs are usually faster, stronger, and connected more effectively on a neurological level (4, 12–17). The asymmetrical division of tasks between the two hemispheres is thought to speed up processing (18). The advantage of the dominant limb is greater, the more difficult the type of movement is, i.e., it is larger for handwriting than for grip strength (19).

A variety of different tests has been used to determine horses' sensory laterality, such as asymmetry of flight responses (20) and investigative behavior (21), their eye preference (20, 22) and emotional reactions (21, 23). Horses that turn to the right can see potentially dangerous stimuli with the preferred left eye. Similar findings exist in other species such as fish, toads, and primates (18, 24–26). Emotionality has correlated to the position of hair whorls (trichoglyphs) in cattle (27, 28) and horses (29–31) in previous studies. Motor laterality has been determined by observing the preferred advanced forelimb during foraging either on pastures (32–35) or with standardized preference tests (36, 37), by documenting the preferred limb for the initiation of movement (38) or truck loading (39), the preferred lead during flat racing (40), as well as the preferred turning side to avoid obstacles (38), the preferred side to roll on (38) and the lateral derailment of the hindquarters while standing (41) or trotting on a circle (42) in foals and young horses. Laterality, as assessed by the riders (43–45) or by experimenters based on judge's scores during competitions (46, 47), has been evaluated. However, other test methods have rarely been investigated for agreement with laterality during riding or among each other, and agreement between studies was limited (36, 38). Especially, with sensory and motor laterality, results varied and relationships between parameters of the two different domains could not be proven in all samples (22, 23, 48). Strong motor laterality does not seem to coincide with strong sensory laterality (48). Even though motor laterality has been observed in foals, too (36, 42), the majority of young horses seem to be ambidextrous (32, 36, 42). Motor laterality in horses might be left-biased (32, 33, 44) based on, e.g., leg preferences for grazing or initiating movement, but right-biased during riding (49–51). Results varied for the main direction of laterality overall (32) and between different breeds such as Standardbreds, Quarter Horses, Thoroughbreds, or feral horses (21, 33, 34).

Besides breed differences and genetic factors, training and handling are frequently suggested as reasons for the increased incidence of laterality in ridden horses (22, 33). However, since motor-biased behavior is present even in foals, training cannot be the only mechanism (6, 22, 36, 41, 42). Similar to human handedness, a combination of genetics and other environmental factors must play a role as well (22, 38, 52).

Although many riding theories call for a predominance of weight and leg aids, in practice, the reins are one of the main means of communication between horses and riders. Furthermore, rein signals are strongly affected by rider handedness (6). Each gait produces a specific pattern of forces applied to the horse's mouth *via* the reins and bit with two spikes per cycle in walk and trot and one spike per stride in canter. These spikes can be explained in relation to the footfall sequences of the gait (53, 54). The mean rein tension reported in different studies varies between the different gaits (walk: 1.3–29.4 N, trot: 3.4–58.8N, canter: 16.2–98 N) (53–57) and different locations and therewith associated training philosophies (58). Asymmetric rein tension has been reported before (56, 59–61) and might lead to negative impacts on the horse's balance, horse-rider communication, and training. Differences in rein contact between the horse's preferred and non-preferred side have been reported in riding theories before (8, 49–51). The results of previous studies indicate that human handedness and horse's laterality might both influence rein tension (56), which is an important measure of horse-rider communication.

The present study aimed to compare the agreement between results obtained by different methods to determine horse's laterality in warmbloods and Thoroughbreds. Furthermore, it aimed at investigating the relation of laterality results obtained outside a riding context to the riders' subjective assessment of the horse's preferred side during dressage tasks and turns and to investigate these results' relation to rein tension measurements.

Since results of a few methods did not agree between and within some earlier studies, it is hypothesized that results might agree between the majority of methods, while other methods are likely to disagree and/or not allow conclusions on laterality during riding.

## Materials and methods

### The different sample populations

The present study included two samples (A, B) of horses, and for each horse, information on age, sex, breed, and coat color was recorded.

Sample A included three groups of warmblood-type horses (warmbloods, riding ponies, and warmblood mixes, *n* = 67, age 0.25–23 years) observed at pasture in order to compare agreement between the results of different methods and their relation to the riders' assessment of laterality as well as to rein tension. The majority (*n* = 46) of horses in this sample were young horses that had not yet been ridden. For the 21 ridden horses (sub-sample A1), laterality was assessed by their riders (all right-handed). Of sub-sample A1, 12 horses (sub-sub-sample A2, age 7–23 years) with 10 right-handed riders were available for rein tension measurements.

Sample B was a group of 61 Thoroughbreds (age 0.003–19 years) observed at pasture in order to compare agreement between results of different methods for laterality assessment obtained outside a riding context as well as between results of the parents and their offspring. The group of horses consisted of one Thoroughbred stud, 13 Thoroughbred broodmares, and their offspring (*n* = 24) from 5 consecutive years. A further 18 horses were either by another stallion or out of a different mare. Additionally, five to at least the third generation unrelated Thoroughbreds were included in the sample.

### Methods to determine the direction of horses' laterality

**Figure 1:**
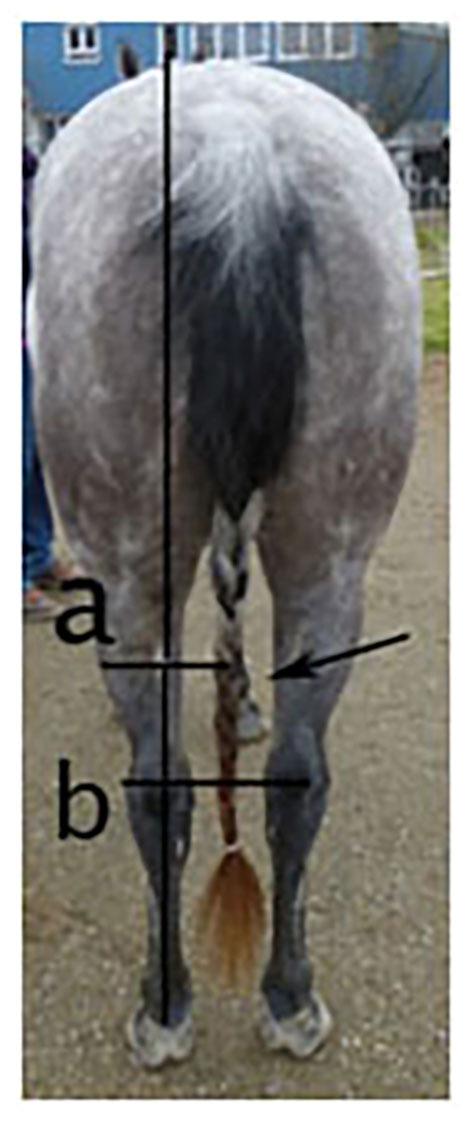
Lateral displacement of the hindquarters to the right with the right forelimb visible between the hind limbs (arrow). Line b shows the position of the hind limbs (hocks). Line a indicates the position of the front limbs (fetlocks). The vertical reference line shows the position of the front fetlocks in relation to the hocks.

**Figure 2:**
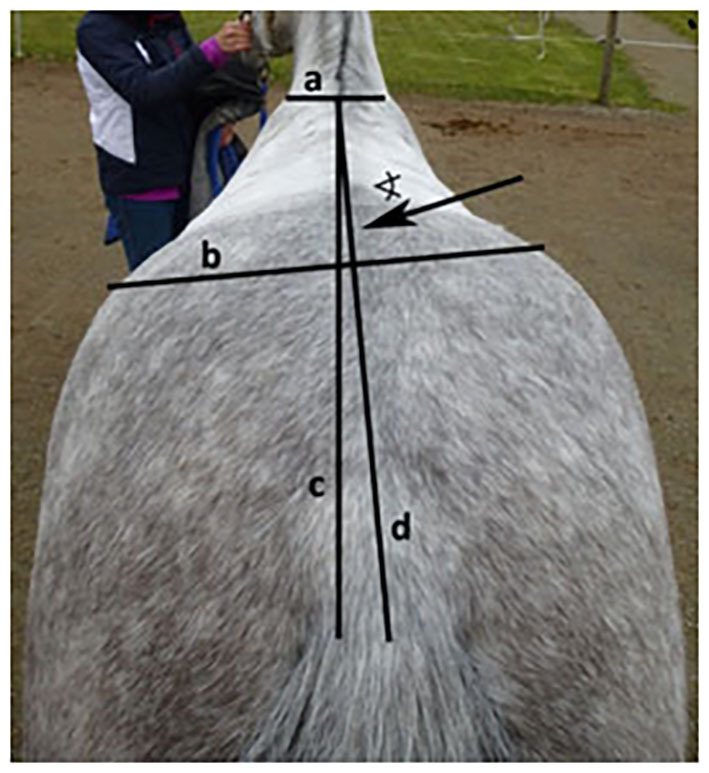
Reference lines to determine the angle of deviation of the spine (line d) from the perpendicular through the withers (line c) in a horse with its hindquarters displaced to the right, using the shoulders (line a) and tuber coxae (line b) as anatomical landmarks.

In the present study, horses were classified according to a variety of established as well as newly developed methods [total *N* = 9 methods; most methods applied to both samples (A, B) of horses], all conducted live:

– (1a) (ForelegGraze30) The advanced foreleg (left or right) during grazing on a pasture was recorded with pen and paper using scan sampling at intervals of 30 s for 2 h as previously described by McGreevy and Rogers (32) (Applied to sample A + B). Pastures were located at different yards and varied in size between 300 and 2,500 m^2^ with 2–6 horses per pasture. Data collection was conducted by one of the authors who was located at the edge of the pasture. To allow the sight of all horses at a given scan, the observer position was slightly adapted, if necessary. Data comprised at least 50 observations with one advanced foreleg per horse. To examine whether a horse showed a significant preference for advancing either the left or right foreleg, a laterality index was used (see Section Statistical analysis).
– (1b) (ForelegGraze60) The advanced foreleg (left or right) during grazing on a pasture was recorded with pen and paper using scan sampling at intervals of 60 s (i.e., counting every other recording of the 30-s samples) for 2 h as described above (Applied to sample A + B). To examine whether a horse showed a significant leg preference, a laterality index was used (see Section Statistical analysis).
– (2) (ForelegBucket) The preferred advanced foreleg (left or right) when feeding carrots from a bucket on the ground was determined in 15 consecutive trials. Horses started the approach to the bucket from different distances (3–20 m) either on a long lead rope or individually in an alleyway as previously described by Van Heel et al. (36) and optimized by Van Dierendonck (37). The requirement for a valid trial was a minimum distance of 28.5 cm (horses) or 25.5 cm (ponies) between the front feet when feeding on the ground as observed from the side of the horse (37). Reference markers on the ground next to the food bucket ensured that the distances were estimated correctly. Since with these methods (1a, 1b, 2), there were multiple observations per horse, a laterality index was calculated (see section Section Statistical analysis) to determine final laterality according to this method [in line with (32, 36, 37)] (Applied to sample A2).
– (3) (EyeNovelObject) The visual laterality (preferred eye for investigation) during the confrontation with 3 novel objects (plastic bag, ball, toy) was determined as previously described by Larose et al. (22) and de Boyer des Roches et al. (23). Horses were frontally approached once with each novel object. The eye that was used first to look at the object was recorded. Since the agreement between results from the three objects was high (see Section Statistical Analysis) a combined rating for visual laterality was created (Applied to sample A + B).
– (4) (FacialHairWhorl) The number and direction (left/right/radial) of facial hair whorls (trichoglyphs) was examined and recorded with paper and pen by an observer standing in front of the horse and classified as previously described by Murphy and Arkins (44); i.e., horses with left-rotating whorls (i.e., hairs growing upright are oriented slightly to the left when viewed facing the horse) were classified as left-lateral, horses with right-rotating whorls as right-lateral and horses with radial whorls or several whorls with opposite directions were classified as ambidextrous (Applied to sample A + B).
– (5) (Mane) The direction of mane (left, right, bilateral as viewed from the horse's perspective) was recorded. All horses with a bilateral (split) mane carried their mane to both sides at the same time and were classified as ambidextrous. Horses with their mane on their left side were classified as left-lateral, and horses with their mane on their right side as right-lateral (Applied to sample A + B).
– (6) (HindquartersLR) As a new approach, the lateral displacement of the hindquarters in relation to the median plane was evaluated from behind at a distance of 2–3 m for each horse while the horse was standing with parallel hind limbs. When viewed from behind, the hindquarters were regarded as a fixed point and the lateral deviation of the shoulders was determined depending on which front limb was visible between the hind limbs. To control the observer's position, both hocks were used as a reference point, which was required to be at an equal distance centrally in front of the observer's feet. As a second step, the deviation of the hindquarters was deduced when regarding the shoulders as a fixed point, i.e., for a deviation of the hindquarters to the right, the right front leg was visible between the hind limbs (Figure 1). If the left front leg was visible between the hind limbs, the horse was classified as left-lateral according to this method. Horses standing entirely square with no front limb visible between their hind limbs were not observed in this sample. This differentiation was chosen according to the theory of “natural crookedness” in the common riding literature (8, 49, 51, 62), which refers to a lateral displacement of the hindquarters (Applied to sample A + B). The repeatability of this method was shown to be high with 79% agreement between repeated assessments of the same horses (*n* = 67) in another study sample (6).
– (7) (HindquartersDegree) In order to determine the degree of the lateral displacement of the hindquarters, a photograph was taken showing the top of the horse's croup, back, and shoulders from behind. Using anatomical landmarks to draw reference lines, the angle of deviation of the horse's spine from a perpendicular through the withers was recorded (Figure 2; applied to sample A2). If the hindquarters were displaced to the right, the values were assigned a negative sign, while values from displacements to the left were assigned a positive sign, resulting in a continuous variable.
– (8) (RiderAssessment) Horse's laterality was assessed by their riders as previously described by Murphy and Arkins (44), i.e., riders were asked to name the preferred or more supple side for dressage tasks and turns, and horses were classified as left-lateral, right-lateral, or ambidextrous accordingly (Applied to sample A1).
– (9) (ReinTensionMean; ReinTensionSD) Rein tension patterns (mean rein tension and mean SD) were investigated for associations between rein tension symmetry and laterality in relation to the direction of movement (clockwise vs. counter-clockwise) as previously described by Kuhnke et al. (56) in order to find out whether differences between asymmetric rein tension of right-handed riders with left- and right-lateral horses can be observed with left-handed riders and different directions of laterality in their horses as well (Applied to sample A2). Data collection took place in indoor and outdoor arenas of various sizes, as horses were stabled at different yards. A rein tension device (Centaur, Netherlands) was fixed between the reins and the bit. The device was calibrated before each test ride. The test ride consisted of 3 circles (20–30 m in diameter) and 3 straight lines (30–60 m long) in each gait (walk, rising trot, sitting trot, and canter) and each direction (clockwise and counter-clockwise), as well as transitions (canter-trot, trot-walk, walk-halt) during conventional European riding. Riders were allowed a warm-up period of 15 min before data collection started. Since each horse–rider pair chose to ride at a speed that suited them best and the arenas varied in size, the total time of data collection for each rider varied. Rein tension was recorded with a frequency of 100 Hz, sent wirelessly to a computer, and recorded with the Centaur software. Each test ride was videotaped, starting with a shot at the computer screen showing the program recording the data in order to ensure the correct assignment of each task to the associated rein tension data. Rein tension parameters (mean, mean SD) were calculated based on the collected data for each rein in each gait and direction.

### Statistical analysis

Statistical analysis was conducted using SAS 9.4 and SPSS 23.0. The sample size was determined for the McNemar Exact Conditional Test to achieve a power of 90% at nominal alpha = 5% for a two-sided test (discordant proportions: 15 and 45%) using the SAS procedure “power”, resulting in a required sample size of 66 horses per individual sample (A, B). Due to the additional availability of one horse in sample A, and due to a shortfall in 5 horses in sample B, actual power was 91% for sample A, 88% for sample B, and 99.6% for the combined sample (A + B). Since all but the riding- and handling-related tests could be conducted in both samples and so could be analyzed as a combined sample, these sample sizes were deemed to be sufficient.

The laterality index (z-value) for method 2 (ForelegBucket) was based on the number of left vs. right advanced forelimbs in relation to the total number of observations (36, 37). A Z-value of ≥1.96 was classified as significant. For EyeNovelObject, there were discrepancies for only *n* = 8 object, and agreement between results from the three objects was substantial to almost perfect (κ = 0.79–0.87; *p* < 0.0001). Therefore, a combined rating for visual laterality was created, such that the respective laterality was assigned, if two out of three objects revealed the same laterality (e.g., two objects were observed with the right eye and one object with both eyes simultaneously or vice versa; *n* = 10) or ambidexterity if all three objects revealed different results (i.e., object 1 using the right eye, object 2 using the left eye and object 3 using both eyes simultaneously; *n* = 1).

Laterality test results yielding categorical data (i.e., all except HindquartersDegree, ReinTensionMean, ReinTensionSD) were tested per sample (A/warmbloods; B/Thoroughbreds) for equal distribution using chi-square tests. If chi-square statistics indicated a significant deviation from an equal distribution among the three categories (left/right/ambidextrous) for a given test method, we additionally tested for a deviation from an equal distribution in individuals classified as left and right only (i.e., omitting ambidextrous individuals) using exact tests for binomial proportions. Bonferroni correction for 8 comparisons/population resulted in a significance level of *p* < 0.00625. Further, crosstabulations of these laterality results with gender (male, female), age class (i.e., foals, yearlings, 2-year-olds, 3-year-olds, 4–5 years, 6–10 years, 11–15 years, and ≥16 years) and coat color (black, bay, gray, chestnut) were used to screen for interrelationships between laterality and these variables. Bonferroni correction for 24 comparisons resulted in a significance level of *p* < 0.0021.

Agreement between any two methods yielding categorical data (i.e., all except HindquartersDegree, ReinTensionMean, ReinTensionSD) was assessed based on simple kappa coefficients and Mc Nemar's test (dichotomous data/left, right) or weighted kappa coefficients and the Bowker's Test of Symmetry (trichotomous data/left, right, ambidextrous). For these pairwise comparisons between the eight methods (*n* = 28 comparisons in total), a Bonferroni correction was applied, resulting in a significance level of *p* < 0.0018. Additionally, sample B test results of the stallion and broodmares of each method were compared to their offspring's test results for agreement.

ReinTensionMean, ReinTensionSD, and HindquartersDegree were assessed for normal distribution and found to be normally distributed (HindquartersDegree: Kolmogorov–Smirnov: *p* > 0.15) or near-normally distributed to justify parametric methods (ReinTensionMean, ReinTensionSD: Kolmogorov–Smirnov: *p* < 0.01). The relationship between HindquartersDegree and all other methods to determine laterality except ReinTensionMean and ReinTensionSD was assessed using an analysis of variance (proc ANOVA) with one other laterality parameter at a time as the sole independent variable.

The relationship between ReinTensionMean and ReinTensionSD (y_i_: mean or mean SD of the i-th observation) and results of other methods were analyzed using mixed models, considering gait (G_j_, with j = walk, rising trot, sitting trot, canter, transitions separately between all gaits and halts), riding direction (D_k_ with k = clockwise, counter-clockwise), task (T_l_ with l = circle, straight line) and rein (Z_m_, with m = left, right) and their interactions as fixed effects, and rider (R_n_), horse (H_o_), and horse^*^rider as random effects in the base model. *e*_*p*_ represents the random error. Laterality according to a given laterality test method (L_q_) alone and in interaction with rein and additional variables (e.g., age as either continuous or categorical variable, horse gender) were consecutively added to the model. The model was reduced again if a term was not significant. Consequently, the model was, e.g.,:


$$\begin{array}{l} y_{\text{ijklmnopq}}=G_{\text{j}}+D_{\text{k}}*T_{\text{l}}*Z_{\text{m}}+L_{\text{q}}R_{n}+H_{o}+H_{n}*R_{o}+e_{p} \end{array}$$

## Results

A significant deviation from an equal distribution was documented for the Thoroughbreds for ForelegGraze30, ForelegGraze60, and EyeNovelObject, although with all three methods the majority of horses were classified as ambidextrous (Table 1), while the proportion of left- and right-lateralized individuals did not deviate significantly from an equal distribution. When considering only lateralized horses, there was a significant left bias at the population level for Thoroughbreds with EyeNovelObject. For warmbloods, significant lateralization at the population level was documented for the method HindquartersRL (majority right-lateral; Table 1). For the methods, EyeNovelObject, ForelegGraze30, ForelegGraze60 as well as Mane, distributions also deviated significantly from an equal distribution, but in all cases, this was caused by either considerably more (all except Mane) or fewer (Mane) ambidextrous horses than expected rather than a strong population bias for either left- or right laterality (Table 1). Except for the two different sampling intervals for the advanced foreleg during grazing (ForelegGraze30 and ForelegGraze60), which significantly agreed with each other (Table 2), for methods conducted outside a riding context, no significant agreement with other methods could be documented (κ not different from 0 and/or *p* > 0.0018; Table 2). However, HindquartersLR showed substantial agreement with RiderAssessment [κ = 0.77 (0.47–1.0); *p* = 0.0003], and RiderAssessment related to ReinTensionMean and ReinTensionSD (Table 2). Age, sex, and coat color did not relate to the results of the laterality tests (all *p* > 0.05). No relationship of lateralized behavior or the direction of laterality between either, the stallion and his offspring, the brood mares and their offspring, or both parents and their offspring was found (*p* > 0.05).

**Table 1:** Overview of the applied laterality test methods and proportions of respective laterality in the different sample populations.

**Method**	**Variable**	**Sample/number of horses**	**% right-lateral**	**% ambidextrous**	**% left-lateral**	**Side preference at the population level**	**Test statistics**
ForelegGraze30 (1a) and ForelegGraze60 (1b)	Foreleg which is significantly more often placed in front	A/67 Warmbloods	1.5 1.5	**91.0** **92.5**	7.5 6.0	Majority of horses ambidextrous	**X**^2^ **=** **100.7;** ***p*** **<** **0.0001;** **X**^2^ **=** **105.9;** ***p*** **<** **0.0001;**
	Z-value ±1.95 = significant	B/61 Thoroughbreds	11.5 8.2	59.0 62.3	29.5 29.5	Majority of horses ambidextrous	**X**^2^ **=** **21.0;** ***p*** **<** **0.0001;** **X**^2^ **=** **27.2;** ***p*** **<** **0.0001**
ForelegBucket (2)	Foreleg which is significantly more often placed in front; Z-value ± 1.95 = significant	A2/12 Warmbloods	0.0	91.7	8.3	Majority of horses ambidextrous	X^2^ = 8.3; *p* = 0.0039
EyeNovelObject (3)	Preferred eye (left, right, both)	A/67 Warmbloods	1.5	83.6	15.9	Majority of horses ambidextrous	**X**^2^ **=** **77.9;** ***p*** **<** **0.0001**
		B/61 Thoroughbreds	4.9	62.3	32.8	Majority of horses are ambidextrous; Significantly more horses left than right-biased	**X**^2^ **=** **30.1;** ***p*** **<** **0.0001; left-right: X**^2^ **=** **12.5;** ***p*** **=** **0.0004**
FacialHairWhorl (4)	Clockwise (right), counter-clockwise (left),	A/67 Warmbloods	20.9	47.8	31.3	Equal distribution of ambidextrous, left- and right lateral horses	X^2^ = 7.4; *p* = 0.0251
	Radial/mismatching double whorls (ambidextrous)	B/61 Thoroughbreds	27.9	32.8	39.3	Equal distribution of ambidextrous, left- and right lateral horses	X^2^ = 1.2; *p* > 0.05
Mane (5)	Left, right, bilateral	A/67 Warmbloods	44.8	1.5	53.7	Fewer ambidextrous horses than expected	**X**^2^ **=** **31.4;** ***p*** **<** **0.001**
		B/61 Thoroughbreds	47.5	34.4	18.0	Equal distribution of ambidextrous, left- and right lateral horses	**X**^2^ **=** **8.0;** ***p*** **=** **0.0183**
HindquartersLR (6)	Displacement to the left or right	A/67 Warmbloods	73.1	0.0	26.9	Right-preference. No ambidextrous horses	**X**^2^ **=** **14.3;** ***p*** **<** **0.0001**
		B/61 Thoroughbreds	60.7	0.0	39.3	Equal distribution of left- and right lateral horses; no ambidextrous horses	X^2^ = 2.8; *p* > 0.05
HindquartersDegree (7)	Degree of lateral displacement	A2/12 Warmbloods	Mean + SD: −0.6°±3.8			The degree of bias is not stronger to the right than to the left	*p* > 0.05
			Min: −7° Max: 5°				
RiderAssessment (8)	Easier to ride in clockwise or counter-clockwise direction or no difference	A1/21 Warmbloods	66.7	0.0	33.3	Equal distribution of left- and right lateral horses; no ambidextrous horses	X^2^ = 2.3; *p* = 0.1266
ReinTensionMean and ReinTensionSD (9)	Rein tension (N, mean and mean SD) in relation to the laterality of horses and riders and the direction of track	A2/12 horse-rider pairs	Mean: right hand: 14.2 ± 1.5 N left hand: 13 ± 1.5 N	Mean: Left-lateral horse: 17.6 ± 2.5 N Right-lateral horse: 9.5 ± 1.5 N		Higher mean tension was applied with the dominant (right) hand and to left-lateral horses (rider's assessment). The magnitude and stability (SD) of mean rein tension varied in relation to the direction of riding and the horse's preferred side.	**Rider:** ***p*** **=** **0.044** **Horse:** ***p*** **=** **0.02** **Stability (SD):** ***p*** **<** **0.0001**

χ^2^-test for all except rein tension parameters and the degree of displacement; additionally exact binomial tests were conducted only for lateralized individuals [left-right; only results significant at *p*_(Bonferroni)_ <0.00625 reported] if there was a significant deviation from an equal distribution between left, right, and ambidextrous individuals; ANOVA (degree of displacement of hindquarters) and mixed model analysis of mean rein tension and SD of rein tension; Bold values are significant results. Grey shade = warmbloods; white/no shade: Thoroughbreds.

**Table 2:** Overview of the agreement between results of the applied laterality test methods across both sample populations [below diagonal (weighted) kappa coefficient and 95% KI; above diagonal: Probability > |Z| based on Mc Nemar's test (dichotomous data) or Bowker's Test of Symmetry (trichotomous data)/F-test for degree of hindquarters and mean + SD of rein tension; significance level (Bonferroni correction) *p* < 0.0018 (28 comparison) and *p* < 0.006 (8 and 9 comparisons) and/or 95% KI includes 0; diagonal: sample size].

	**Advanced foreleg during grazing 30 s scan sampling**	**Advanced foreleg during grazing 60 s scan sampling**	**Advanced foreleg during feeding from a bucket (limb preference test)**	**Visual laterality (novel object test)**	**Direction of facial hair whorls (trichoglyphs)**	**Direction of mane**	**Lateral displacement of the hindquarters in relation to the median plane while standing**	**Degree of the lateral displacement of hindquarters**	**Rider's assessment (preferred side for dressage tasks)**	**Rein tension symmetry**
Advanced foreleg during grazing 30 s scan sampling	*n* = 128	***p*** **<** **0.0001**	*p* > 0.0018	*p* > 0.0018	*p* > 0.0018	*p* > 0.0018	*p* > 0.0018	*p* > 0.006	*p* > 0.006	Insufficient variance in foreleg laterality in this sub-sample
Advanced foreleg during grazing 60 s scan sampling	**κ** **=** **0.79 (0.66–0.91)**	*n* = 128	*p* > 0.0018	*p* > 0.0018	*p* > 0.0018	*p* > 0.0018	*p* > 0.0018	*p* > 0.006	*p* > 0.006	Insufficient variance in foreleg laterality in this sub-sample
Advanced foreleg during feeding from a bucket (limb preference test)			*n* = 12	*p* > 0.0018	*p* > 0.0018	*p* > 0.0018	*p* > 0.0018	*p* > 0.006	*p* > 0.006	Insufficient variance in foreleg laterality in this sub-sample
Visual laterality (novel object test)				*n* = 128	*p* = 0.0048	*p* = 0.0004	*p* > 0.0018	*p* > 0.006	*p* > 0.006	*p* > 0.006
Direction of facial hair whorls (trichoglyphs)				κ = 0.13 (0.004–0.26)	*n* = 128	*p* > 0.0018	*p* > 0.0018	*p* > 0.006	*p* > 0.006	*p* > 0.006
Direction of mane				κ = −0.06 (−0.14–0.02)		*n* = 128	*p* > 0.0018	*p* > 0.006	*p* > 0.006	*p* > 0.006
Lateral displacement of the hindquarters in relation to the median plane while standing							*n* = 128	*p* > 0.006	***p*** **=** **0.0003**	**Mean:** ***p*** **=** **0.03; SD:** ***p*** **=** **0.0128, interaction with rein-side: mean:** ***p*** **=** **0.0003; SD:** ***p*** **=** **0.0043**
Degree of the lateral displacement of hindquarters								*n* = 11	*p* > 0.006	*p* > 0.006
Rider's assessment (preferred side for dressage tasks)							**κ** **=** **0.77 (0.47–1.00)**		*n* = 21	**Mean:** ***p*** **=** **0.03; SD:** ***p*** **=** **0.0128, interaction with rein-side: mean:** ***p*** **=** **0.0003; SD:** ***p*** **=** **0.0043**
Rein tension symmetry										*n* = 12

Bold values indicate statistically significant results.

### The preferred forelimb during grazing in 30 vs. 60-s scan sampling (ForelegGraze 30 vs. ForelegGraze60)

**Figure 3:**
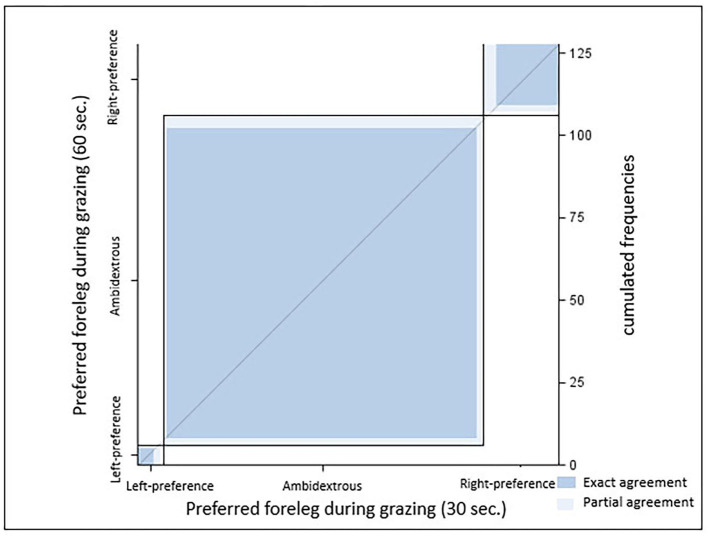
Agreement between leg preference recorded with 30 and 60 s intervals [κ = 0.79 (0.66–0.91), *p* < 0.0001; both samples, *n* = 128 horses]. Dark blue, exact agreement; light blue, partial agreement.

Agreement between results from the two sampling intervals was substantial [κ = 0.79 (0.66–0.91), *p* < 0.0001]. In the 30 s scan sampling, significant (z-value > ±1.96) leg preference was documented in 6 warmbloods (sample A), while 91% did not prefer any leg (X^2^ = 105.9; *p* < 0.0001). Results changed slightly with the 60 s sampling: Only five horses displayed significant leg preference. No leg preference was recorded for 92.5% of sample A (X^2^ = 105.9; *p* < 0.0001). For the individual horses, the associated significance levels remained, increased, or decreased between both samples but never changed direction (*p* < 0.0001, Figure 3). Neither ForelegGraze30 nor ForelegGraze60 showed significant agreement with results from any other laterality test (*p* > 0.0018, Table 2).

With both sampling intervals, the majority of Thoroughbreds showed no leg preference (ForelegGraze30: 59%, X^2^ = 21.0 *p* < 0.0001; ForelegGraze60: 62.3%, X^2^ = 21.0 *p* < 0.0001). Most lateralized horses preferred their left foreleg (29.5% in each interval, *p* < 0.0001). Direction and degree of laterality remained constant in most cases between both sampling intervals.

### Visual laterality (EyeNovelObject)

EyeNovelObject slightly agreed with FacialHairWhorl [κ = 0.13 (0.004–0.26), *p* = 0.0048], but after application of the Bonferroni-correction, this agreement was no longer significant. During frontal approach with novel objects (plastic bag, toy, ball), most horses of sample B did not use a specific eye to look at an object and thus showed no eye preference (62.3%, X^2^ = 30.1; *p* < 0.0001), but of those that used a specific eye, a significant majority preferred the left eye (X^2^ = 12.6; *p* = 0.0004).

### The lateral displacement of the hindquarters (HindquartersLR)

The majority of horses in sample A (73.1%) showed a right-displacement of their hindquarters. Only 26.9% had their hindquarters displaced to the left (X^2^ = 14.3; *p* < 0.0001). In sample B, likewise, all horses had their hindquarters displaced either to the right (60.7%) or to the left (39.3%), however, the bias was not significant at the population level (X^2^ = 2.8; *p* > 0.00625 after Bonferroni correction).

### The lateral displacement of the hindquarters (HindquartersLR) and the rider's assessment of their horse's laterality (RiderAssessment)

**Figure 4:**
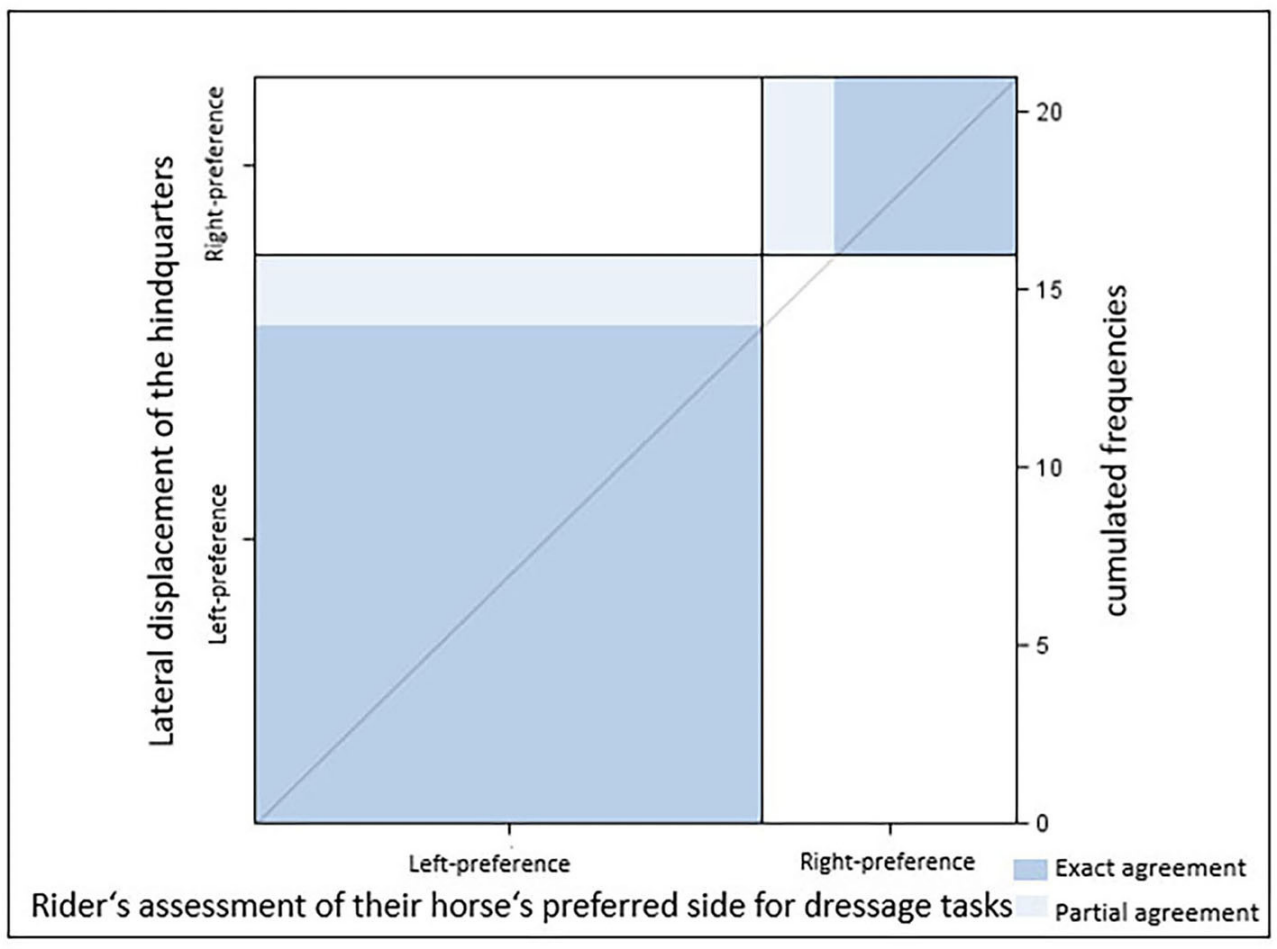
Agreement between the direction of the lateral displacement of the hindquarters and the rider's assessment of their horse's preferred side for dressage tasks [κ = 0.77 (0.47–1.00), *p* = 0.0003; Sample A.1 *n* = 21 horses]. Dark blue, exact agreement; light blue, partial agreement.

There was substantial agreement between HindquartersLR and RiderAssessment [κ = 0.77 (0.47–1.00), *p* = 0.0003; Table 2]. Most ridden horses (sample A1, *N* = 21) with their hindquarters displaced to the right (*n* = 14) were classified as right-lateral by their riders. In some cases (*n* = 2), however, horses with a right displacement of their hindquarter were described as left-lateral. Riders classified all horses with their hindquarters displaced to the left (*n* = 7) as left-lateral (*p* = 0.003, Figure 4). All horses perceived as right-lateral by their riders had their hindquarters displaced to the right (Table 2).

### Rein tension (ReinTensionMean, ReinTensionSD) in relation to other methods to determine laterality

**Figure 5:**
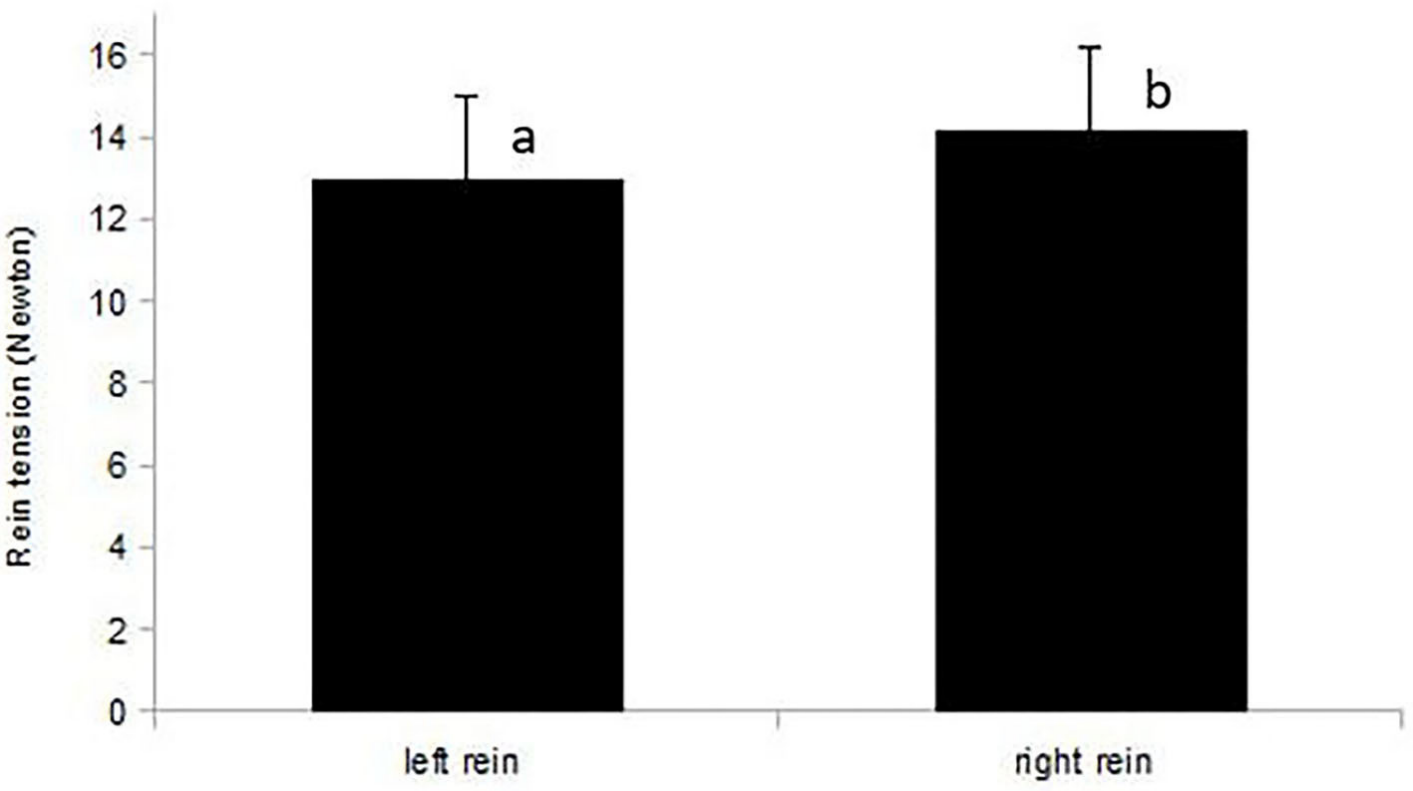
Mean rein tension (N) of the left and right rein in right-handed riders (*n* = 12 rides). Riders applied higher mean rein tension with their dominant, right hand (*p* = 0.044). Different letters indicate a significant difference at *p* < 0.05.

**Figure 6:**
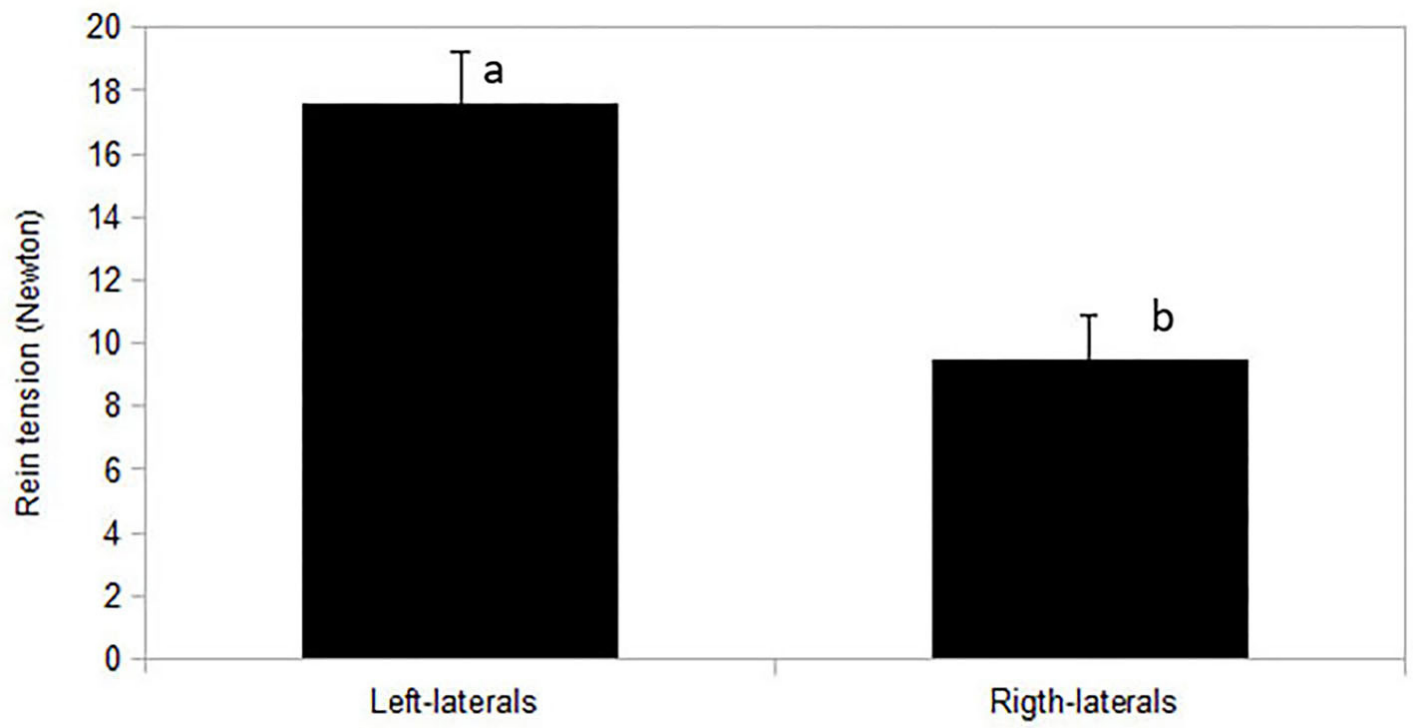
Mean rein tension (N) of both reins overall during riding of a standardized dressage task in relation to horse's laterality (right-lateral *n* = 10, left-lateral *n* = 2) assessed by the riders. Mean of both reins was higher in left-lateral horses (*p* = 0.02). Different letters indicate a significant difference at *p* < 0.05.

For the sample of 12 horses with rein tension analysis [sample A2; 10 right-lateral, 2 left-lateral based on RiderAssessment)], no relation between ReinTensionMean or ReinTensionSD and ForelegGraze30, ForelegGraze60, ForelegBucket, and EyeNovelObject was found (F-test, all *p* > 0.00625). However, asymmetric rein tension patterns (ReinTensionMean) agreed with RiderAssessment (*p* = 0.019) and seemed to be influenced by the laterality of both, horses and riders. Riders (all right-handed) applied higher mean rein tension with their dominant hand (14.2 ± 1.5 N right hand vs. 13 ± 1.5 N left hand, *p* = 0.044; Figure 5). Mean rein tension applied to the left-lateral horses (RiderAssessment) was considerably higher than in right-lateral horses (RiderAssessment) (mean of both reins: 17.6 ± 2.5 N LL vs. 9.5 ± 1.5 N RL, *p* = 0.02, Figure 6).

The difference between tension in the left and right rein was higher when riding in direction of the riders' non-dominant hand (counter-clockwise) with all horses (*p* = 0.02). Rein tension was more stable (i.e., lower ReinTensionSD) when riding in the direction of the riders' dominant hand (clockwise; *p* < 0.0001).

Also, rein tension tended to be higher in horses with their hindquarters displaced to the left (18.57 ± 4.6 N vs. 9.77 ± 2.3 N, *p* = 0.077).

## Discussion

### The direction of mane and FacialHairWhorls

With most Warmbloods in sample A, the mane fell to either the left or right side, and the side of the mane is commonly suggested to be a possible indicator of laterality during riding in horses. However, neither in the present sample nor in other research (47) there was significant agreement between the direction of mane and any other method to determine laterality. Like with facial hair whorls, the side of the mane is determined by the orientation of the hair follicles in the crest. In previous research, a relationship between laterality during riding and the direction of facial hair whorls has been documented (43–45). In other studies, however, these methods did not agree (63). In the present study, the direction of facial hair whorls was equally distributed and did not agree with the results of any other method. One could argue that the sample of ridden horses with known laterality as assessed by their riders might have been quite small (*n* = 21). However, even this rather small sample of horses displayed a clear side bias that could be documented with several other methods such as ReinTensionMean, ReinTensionSD, and HindquartersLR.

### The preferred forelimb during grazing and feeding from a bucket (ForelgGraze30, ForelegGraze60, ForelegBucket)

The preferred advanced forelimb is one of the most common methods to determine horses' laterality (32, 33, 35). As a scan sampling test, 60-s intervals have been applied in most cases. In order to test possible variations in results with different intervals, in the present study, scan sampling has been applied for 30 s intervals (ForelegGraze30), plus counting only every other value to receive a 60-s interval sampling (ForelegGraze60). A significant side bias was detected for individual horses with both intervals. Since the agreement between results of both sampling intervals was substantial and neither method showed a significant relationship to any other methods to determine laterality, it is suggested that when working under time constraints the 60-s intervals could be chosen when using this method to determine the horse's laterality. The lack of agreement between the methods documented during feeding at pasture (ForelegGraze30, ForelegGraze60) and from a bucket (ForelegBucket) is surprising, given the similarity of the methods. However, in the present study, only one horse observed in the bucket test showed a significantly lateralized behavior, and the sample size for the bucket test was too small, to draw definite conclusions. Thus, an agreement between observations taken from feeding at pasture vs. from a bucket should be re-assessed in future studies.

Rider's assessment of their horse's laterality did not agree significantly with any particular leg preference (ForelegGraze30, ForelegGraze60, ForelegBucket). One could argue that the purpose of the limbs during grazing is to support the horse's weight and allow the horse to reach food. The different requirements compared to locomotion whilst keeping their balance during riding might explain the lack of agreement between these methods.

### Visual laterality (EyeNovelObject)

Visual laterality was not related to any other method applied in the present study. While the majority of young Thoroughbreds of sample B were ambidextrous, the remaining lateralized horses were more likely to show a biased reaction to the left side during the investigation of the novel objects. Breeds such as Thoroughbreds have been identified as more emotionally reactive and fearful (22, 64). The use of the left eye correlates with negative emotions, about novel objects or objects that are associated with fearful situations (20–23). A lack of lateralized behavior based on eye preference has been reported by Larose et al. (22) for some horses, too. A possible explanation might be that some horses are not sensory biased or only show a sensory biased reaction when the object triggers a strong emotional reaction. Possibly, the test-setup with the chosen objects and/or a person carrying the novel object lowered the chances of a strong fear reaction, as the person in close proximity to the novel object might have signaled the horses that the objects are nothing to be afraid of. Since the majority of horses in sample B were younger than the horses in sample A, and Thoroughbreds might be more prone to show fear compared to warmbloods, this could explain the greater number of left-biased reactions in the Thoroughbred sample.

The lack of agreement between visual laterality (EyeNovelObject) and tests to investigate motor laterality (e.g., ForelegGraze60) suggests that different types of laterality developed in horses on different levels. In fact, simple motor tasks were found to be most representative of lateralization, while in rather complex tasks such as visual processing asymmetric reactions were strongly influenced by the stimulus (65). Therefore, tests based on sensory laterality EyeNovelObject, do not seem to be useful to conclude on motor laterality. Similar results have been reported earlier (32, 48).

### The lateral displacement of the hindquarters from the median plane (HindquartersLR and HindquartersDegree)

Most of the warmbloods had their hindquarters displaced to the right (Table 1). This result is comparable to the observations of many experts on equitation throughout the centuries (7, 8, 49, 51, 62). In previous scientific studies applying a variety of test methods, laterality was absent or directions of sensory and motor laterality did not agree between individual horses or between different horse breeds (20, 22, 32, 36, 38). However, except for the direction of facial hair whorls, no other method had been scientifically compared to the rider's assessment of laterality during the ride. Evaluating the HindquartersLR or HindquartersDegree, no horse was entirely straight; therefore, both sample A and sample B contained only right- and left-lateral horses. Therefore, both directions of laterality (deviation to the left and right) exist in horses on an individual level. However, one direction of laterality is more frequent at the population level (e.g., right-laterality based on the lateral displacement of the hindquarters to the right in the warmblood population, even though some individual warmbloods showed a lateral displacement to the left). The lateral displacement of the hindquarters has been categorized as a morphological asymmetry, which is supposed to develop before birth and be caused by differences in muscle length (7, 8, 66). Since it is already present even in foals (6, 41, 42), training and a possible influence of the rider's handedness cannot be the only mechanism for its development. Genetics and other environmental factors have been suggested to influence the development of asymmetries as well (22, 38, 52).

Horses with their hindquarters displaced to the right deviated not significantly stronger from the median plane than horses with their hindquarters displaced to the left. Possibly, unlike humans (67, 68), right-lateral horses according to HinquartersRL are not stronger lateralized than left-lateral horses, but this needs to be confirmed in a larger sample of horses since HindquartersDegree could only be assessed in sample A2.

### The lateral displacement of the hindquarters (HindquartersLR) and the rider's assessment of their horse's laterality (RiderAssessment)

The lateral displacement of the hindquarters is easy to assess and clearly visible in all horses. Slightly different approaches to determining the lateral displacement of the hindquarters in other studies showed variation between the horses of the respective samples (41, 42). According to the results of the present study, the lateral displacement of the hindquarters seems to be a reliable indicator of laterality during riding. This relationship has in the meantime been verified in a larger sample [*n* = 106 observations with assessments performed by both right- and left-handed riders, (6)] since for RiderAssessment there were only *n* = 21 observations in the present sample. In that study, HindquartersLR mostly agreed with RiderAssessment regardless of the rider's handedness (6). Taken together, these results suggest that HindquartersLR more so than any other previously tested method is a suitable indicator for the rider's assessment of her/his horse's laterality during riding. However, mixed results were found exclusively in a small number of left-lateral horses (rider's assessment) with their hindquarters displaced to the right. Further investigations of the lateral displacement of the hindquarters according to laterality during riding and the muscular system are required to investigate whether mixed results might be a hint toward a less lateralized or ambidextrous individual.

### Riders' assessment of their horse's laterality (RiderAssessment) compared to rein tension (ReinTensionMean, ReinTensionSD)

There appears to be a strong influence of the rider's dominant hand on the rein tension and the symmetry of the outside vs. the inside rein between both directions, which we observed in a previous study, too (56). The RiderAssessment seemed to be related to the magnitude and symmetry of rein tension [verified in a larger sample with *n* = 106 observations including right- and left-handed riders, (6)]. As previously reported (56), higher rein tension was applied to left-lateral horses by their right-handed riders throughout. A higher magnitude of rein tension was also observed in horses with a displacement of the hindquarters to the left. According to the results of the present sample, symmetry and stability of rein tension seem to be related to the magnitude of mean rein tension and the horse's preferred side. Thus, a symmetric and stable rein contact seemed to be easier to achieve with the right-lateral horses (showing the same side-preference as their riders) as they were ridden with lower mean rein tension. Riding counter-clockwise, their non-preferred left side, which is associated with less symmetric and less stable rein tension patterns and their rider's dominant right hand with stronger rein tension meets the demands of the riding literature to ride with more contact (i.e., higher tension) on the outside rein (8, 49, 62). In a clockwise direction, the rider's dominant hand applies stronger rein tension to the inside rein, thus making rein tension of the inside and outside rein appear almost equal in contrast to demands of the riding literature. However, since this occurs on the horse's dominant side, i.e., the side on which dressage tasks and turns are easier to ride, the horse seems to compensate for the rider's mismatching rein tension signals for the same task in both directions. On the other hand, the theories and demands of the riding literature have been established based on the subjective experiences and impressions of the old riding masters [e.g., (7)]. Both handedness and laterality influence rein tension and riders are often unaware of the magnitude and symmetry of their rein tension even on a model horse (59, 69). The demand to ride with stronger contact on the outside rein could therefore also be based on a compensatory mechanism (i.e., the rider trying to compensate for the horse's attempts to lean on one rein whilst being unaware of their own asymmetric grip strength) that has been perceived to support horse–rider communication but is actually an unclear signal and counterproductive for horse learning.

### The relation of age, sex, and coat color with laterality

Laterality is proven to vary between horses of different age groups and changes from one preference to the other during maturation have been observed (32, 33, 36, 50). Therefore, a large sample containing horses of all ages was chosen. However, with neither of the samples, a relation of age to results of any laterality method or rein tension could be identified in the present study. For some studies with smaller sample sizes, laterality seemed to be biased by sex (38). However, in the current study, this was found for only one test method exclusively in the sample of Thoroughbreds (sample B, results not reported), so it is open to debate whether this result was obtained purely by chance. Similar to the results of sample B, in a previous study, male horses of other sample populations exhibited mostly left-biased behavior, in contrast to female horses which were reported to show a bias to the right most often (38). In the present study, however, female horses showed mostly no laterality or a left-bias, which differs from previous reports for female horses (38). This could be either due to young age, the left bias in the total population of the breed, or possibly due to the direction of laterality of their parents. The sire of most of the young horses, as well as some of the dams, showed a left bias throughout all test methods. In humans, left-handed parents, especially females, are more likely to have a left-handed child (70, 71). However, the mechanism behind the inheritance of left-handedness remains unclear (72) and environmental factors most likely play a role as well (73). In horses, a genetic predetermination might be possible that appears and increases with age and might also be influenced by environmental factors (38). Recent quantitative genetic studies suggest high heritability for laterality based on the lateral displacement of the hindquarters in warmbloods, but low to moderate heritability in Thoroughbreds (74), implying that environmental factors play a large role in the determination of laterality, especially in Thoroughbreds. The fact that in the present study the occurrence and direction of laterality of the parents in the Thoroughbreds of sample B did not significantly relate to their offspring's laterality further supports the rejection of the hypothesis of either genetic influences being the sole or largest trigger for laterality.

A possible explanation for breed differences of lateralized behavior was suggested to be training (33), i.e., especially the influence of the rider (e.g., due to his/her handedness) and/or daily routines that are carried out from the same side repeatedly such as mounting. Furthermore, the selection of horses for different purposes might have led to more symmetrical horses being chosen, e.g., reproduction for dressage than for flat racing, where speed is the most important trait for selection.

## Conclusion

Laterality was documented on a population basis only for the lateral displacement of hindquarters in warmblood-type horses. With most tests, the majority of individual horses showed no side preference and thus the population seems to be ambidextrous for these specific tests. Except for the lateral displacement of the hindquarters, none of the methods documenting laterality outside a riding context allowed conclusions on laterality during riding. Different levels of laterality—even within the area of motor laterality- seem to exist, that are not necessarily related to each other. Thus, the agreement between different aspects of laterality in horses seems to be limited to specific measures and outcomes. Attention should be paid to the desired information when selecting methods for the assessment of laterality. In particular, laterality test results obtained outside a riding context do not appear to predict laterality during riding. The lateral displacement of the hindquarters can give a hint on the direction of laterality during riding for the majority, but not all horses. Only the riders' assessment of their horse's laterality and the lateral displacement of the hindquarters agreed significantly with the laterality patterns of rein tension. A horses' laterality has an impact on the magnitude and symmetry of rein tension. Horse-rider combinations with the same direction of laterality seemed to be better coordinated and showed a lighter rein contact. Matching horses and riders according to their laterality might be beneficial for the symmetry of rein tension and thus improve training.

## Data availability statement

The datasets presented in this article are not readily available because data is part of consecutive research. Requests to access the datasets should be directed to SK, s.kuhnke@arcor.de. Data will be available upon reasonable request.

## Ethics statement

Ethical review and approval was not required for the study on human participants in accordance with the local legislation and institutional requirements. The patients/participants provided their written informed consent to participate in this study. Ethical review and approval was not required for the animal study because this type of non-invasive study did not require a study-specific license for ethics/animal welfare approval at the time when the study was conducted (2013–2015). Written informed consent was obtained from the owners for the participation of their animals in this study.

## Author contributions

SK and UK conceived the idea of the paper, designed the experiments, analyzed the data and wrote the paper. SK performed the experiments. Both authors contributed to the article and approved the submitted version.

## Funding

Article publication was supported by the Open Access Publication Fund of the Justus-Liebig University of Giessen.

## Conflict of interest

The authors declare that the research was conducted in the absence of any commercial or financial relationships that could be construed as a potential conflict of interest.

## Publisher's note

All claims expressed in this article are solely those of the authors and do not necessarily represent those of their affiliated organizations, or those of the publisher, the editors and the reviewers. Any product that may be evaluated in this article, or claim that may be made by its manufacturer, is not guaranteed or endorsed by the publisher.
